# The Association between Systemic Inflammatory Cellular Levels and Lung Function: A Population-Based Study

**DOI:** 10.1371/journal.pone.0021593

**Published:** 2011-07-20

**Authors:** Tricia McKeever, Shiron Saha, Andrew W. Fogarty

**Affiliations:** 1 Nottingham Respiratory Biomedical Research Unit, Division of Epidemiology and Public Health, University of Nottingham, City Hospital, Nottingham, United Kingdom; 2 Department of Respiratory Medicine, Sheffield Teaching Hospitals NHS Trust, Northern General Hospital, Sheffield, United Kingdom; Ludwig-Maximilians-Universität München, Germany

## Abstract

**Background:**

Lower lung function is associated with an elevated systemic white cell count in men. However, these observations have not been demonstrated in a representative population that includes females and may be susceptible to confounding by recent airway infections or recent cigarette smoking. We tested the hypothesis that lung function is inversely associated with systemic white cell count in a population-based study.

**Methods:**

The study population consisted adults aged 17−90+ years who participated in the Third National Health and Nutrition Examination Survey who did not report a recent cough, cold or acute illness in a non-smoking and smoking population.

**Results:**

In non-smoking adults with the highest quintile of the total white cell count had a FEV_1_ 125.3 ml lower than those in the lowest quintile (95% confidence interval CI: −163.1 to –87.5). Adults with the highest quintile of the total white cell count had a FVC 151.1 ml lower than those in the lowest quintile (95% confidence interval CI: −195.0 to −107.2). Similar associations were observed for granulocytes, mononuclear cells and lymphocytes. In current smokers, similar smaller associations observed for total white cell count, granulocytes and mononuclear cells.

**Conclusions:**

Systemic cellular inflammation levels are inversely associated with lung function in a population of both non-smokers and smokers without acute illnesses. This may contribute to the increased mortality observed in individuals with a higher baseline white cell count.

## Introduction

Chronic obstructive pulmonary disease (COPD) is a common disease characterised by reduced lung function and obstructive airflow limitation [1]. Over the past decade there has been increasing interest in the potential importance of systemic inflammation, both with regard to the pathogenesis of COPD [2]–[4] and also as a biomarker to study associations between lung function and systemic inflammation in population-based studies [5]-[8]. The total white blood cell count is an established measure of the systemic inflammatory response and surveys in the 1980s [9]–[11] first reported an inverse association between Forced Expiratory Volume (FEV_1_) and total white cell count. However, these data only studied men and are susceptible to confounding by recent airway infection [12] or incomplete adjustment for cigarette smoking.

We have used data from the Third National Health and Nutrition Examination Survey (NHANES III) to establish a population of individuals who do not currently smoke cigarettes and also a population of current smokers who were free of recent respiratory and acute illness. We investigated the relationship between the constituents of the white blood cell count and lung function as measured by FEV_1_ and FVC. We hypothesised that an increase in the cellular systemic inflammation levels as measured by the total white blood cell count and its constituent cells would be associated with a reduction in both FEV_1_ and FVC.

## Methods

### Study population

#### Ethics statement

The study received approval from a Research Ethics Review Board [13] and all participants gave their written consent prior to participation in the study. We used data from NHANES III, a survey designed to examine the health and nutrition of a randomly selected sample of the US population. The survey was conducted between 1988 and 1994 and full details of the survey design and examination procedure have been published by the National Centre for Health Statistics [14]. The study received approval from a Research Ethics Review Board and all participants gave their consent prior to participation in the study. The population studied were aged over 17 years. To remove the potential confounding factor of recent illness on lung function or systemic white cell count, individuals/subjects were excluded if they responded ‘yes’ or ‘unknown’ to the question ‘in the past few days have you had a cough, cold, or other acute illness’. There were 2 study populations, the first population were self-reported non-smokers who also had a cotinine value of less than 15 ng/ml [15] and the second consisted of self-reported current smokers. Exclusions were also made for missing data on the total white cell count, lung function, smoking status and body mass index.

### Data collection

Blood samples were taken for measurement of cotinine, the total white cell count and its constituent differential cells including granulocytes, lymphocytes, monocytes, eosinophils and basophils. White blood cell count was determined using a fully automated Coulter S-PLUS JR hematology analyzer (Beckman Coulter, Fullerton, California, United States). Anthropometric measurements were made including height, weight and spirometry using standardized techniques (including FEV_1_ and FVC). A detailed description of laboratory methods has been published [16].

### Statistical analyses

Using self-reported smoking history we quantified total cigarette consumption in pack-years. Lung function measures were modelled adjusting for age, sex, height, smoking (status and pack-years), body mass index (BMI) and race/ethnicity to derive the most parsimonious model that minimized variance. More complex models for lung function of higher order variables for age and height were examined but these did not improve the fit of the model, and therefore the simplest model was chosen. Inflammatory cell counts were arbitrarily categorized into quintiles separately in non-smoking and smoking populations and the relationships between measures of these and lung function were explored using multivariate linear regression. We fitted the models with quintiles of inflammatory cell counts as both ordered and unordered factors and compared the two models for evidence of departure from linearity. The p-value for linear trend was given when there was no departure from linearity. The results are presented in quintiles and we also present the data as per SD increase of the inflammatory cell count as all associations observed were linear. Potential confounders such as plasma glucose and waist:hip ratio were fitted as continuous or categorical variables as appropriate, and were examined to determine whether they altered the regression coefficients. A factor was considered a confounding factor if its addition to the model changed the size of effect by 15% or more. A sensitivity analyses was conducted excluding individuals with greater than 10 cigarette pack years and only including individuals with a serum cotinine <3 ng/ml [17] (which represents lower levels of exposure to environmental tobacco smoke). The data was also investigated to determine if the association differed according to gender.

Due to complex, multistage probability sample design of NHANES III, estimates were calculated accounting for the survey design, using the specialized survey command within STATA SE 10.0 (Stata Corporation, Texas).

## Results

From the total study population of 20050 individuals aged 17−90+ years who provided data for the NHANES III survey, 4990 participants reported current cigarette consumption and of those who did not smoke, 3163 participants either reported a cough, cold, or other acute illness in the previous few days or did not declare the status of these symptoms. This gave a study population of 11867 individuals of whom 8381 participants had cotinine of <15 mg/ml and provided paired data permitting assessment of the association between white cell count and lung function while also adjusting for possible confounding factors. The study population is described in Table 1. Using similar selection criteria for those participants who were current smokers, 2790 individuals met the study inclusion criteria (Table 1).

**Table 1:** Demographics and characteristics of the study population of individuals who have never smoked selected from the adult NHANES III population*.

Variable	Non-Smoking PopulationN = 8381		Current smokersN = 2790	
	Mean (SD)	Number (%)	Mean (SD)	Number (%)
**Sex**MalesFemales		3579 (42.7)4802 (57.3)		1603 (57.5)1187 (42.5)
**Age**	46.4 (20.4)		41.0 (16.1)	
**Smoking status**NeverExCurent		5972 (71.3)2409 (28.7)		2790 (100)
**Pack years** **	14 (3.5 to 36)		11 (3.2 to 25.5)	
Race/EthnicityNon-Hispanic WhiteNon-Hispanic BlackMexican-AmericanOther		3485 (41.6)1990 (23.7)2514 (30.0)392 (4.7)		1061 (38.0)933 (33.4)701 (25.1)95 (3.4)
**Height (cm)**	165.7 (9.9)		168.8 (9.2)	
**BMI (kg/m^2^)**	27.2 (5.8)		25.9 (5.4)	
**WHR (n = 9008)**	0.91 (0.09)		0.92 (0.09)	
**FEV_1_ (ml)**	2970 (943)		3144 (943)	
**FVC (ml)**	3719 (1103)		4013 (1071)	
**FEV_1_/FVC**	0.80 (0.09)		0.78 (1.0)	
**Asthma** **		502 (6.0)		188 (6.7)
**COPD** **		697 (8.7)		397 (14.7)
**White cell counts**				
**Total white cells x10^3^**	6.91 (2.13)n = 8381		7.9 (2.3)n = 2790	
**Granulocytes x10^3^**	4.27 (1.63)n = 8256		4.88 (1.89)n = 2732	
**Mononuclear cells x10^9^/l**	0.40 (0.21)n = 8256		0.45 (0.23)n = 2732	
**Lymphocytes x10^3^**	2.24 (0.97)n = 8380		2.52 (0.81)n = 2789	
**Eosinophils** ***	2 (1–4) SD = 2.7n = 2260		2 (1–4) SD = 2.7n = 798	
**Basophils** ***	0 (0–1)SD = 0.7n = 2269		0(0–1)SD = 0.8 n = 798	

*study population excludes individuals who report a cough, cold or other acute illness in the past few days.

**Asthma as defined by self report of doctor diagnoses and COPD defined by as self-reported physician-diagnosed emphysema and/or chronic bronchitis, or by GOLD spirometry criteria (FEV_1_/FVC<70% and FEV_1_<80%,although post bronchodilator values were unavailable).

***median and interquartile range.

The total white cell count was inversely associated with FEV_1_ with the highest quintile being −125.3 ml (95% confidence intervals CI: −163.1 to −87.5) lower than the lowest quintile (Table 2). Similar inverse associations were seen for granulocytes (highest vs lowest quintile −119.3 ml; 95%CI: −156.7 to −81.9), mononuclear cells (highest vs lowest quintile −95.1 ml; 95%CI: −130.2 to −60.1) and lymphocytes (highest vs lowest quintile −50.6 ml; 95%CI: −81.3 to −19.9). There was no association between FEV_1_ and eosinophils and basophils. Similar findings were found in relation to FVC, such that the total white cell count was also inversely associated with FVC (highest vs lowest quintile

**Table 2:** Association between categories of blood inflammatory cells and Forced Expiratory Volume in one second in non-smoking adults*.

White cell category	Values	Forced Expiratory Volume in one second**	
		Coef(ml)	95% CI
**Total white cell count (n = 8351)**	1.75–5.255.26–6.26.21–7.157.16–8.358.36–43.65per SD increase	0−23.8−39.6−82.1−125.3p-trend <0.001−45.0	−56.9 to +9.3−71.8 to −7.5−112.7 to −51.5−163.1 to −87.5−56.4 to −33.6
**Granulocytes (n = 8256)**	0.2–2.952.96–3.73.71–4.44.41–5.45.41–17.75per SD increase	0−18.9−41.8−75.5−119.3p-trend <0.001−41.3	−44.3 to +6.4−74.2 to −9.4−106.9 to −44.2−156.7 to −81.9−53.1 to −29.5
**Mononuclear cells (n = 8256)**	0–0.250.26–0.350.36–0.40.41–0.50.51–5.35per SD increase	0−7.3−20.1−57.7−95.1p-value <0.001−38.1	−33.1 to +18.5−56.4 to +16.3−92.9 to −22.5−130.2 to −60.1−49.8 to −26.4
**Lymphocytes (n = 8380)**	0.35–1.651.66–1.951.96–2.32.31–2.752.76–33.85per SD increase	010.03.1−15.7−50.6p-trend = 0.001−17.7	−23.0 to 42.9−31.8 to 37.9−50.5 to 19.1−81.3 to −19.9−25.8 to −9.6
**Eosinophils (n = 2260)**	0–123–45–25per SD increase	08.8−33.5−43.5p-trend = 0.14−16.7	−47.7 to 65.3−85.3 to 18.3−107.8 to 20.8−39.2 to 5.8
**Basophils (n = 2260)**	012–6per SD increase	026.02.2p-trend = 0.385.24	−12.5 to 64.5−61.4 to 65.8−13.9 to 24.4

*study population excludes individuals who report a cough, cold or other acute illness in the past few days.

**adjusted for age, sex, height (continuous), race/ethnicity, body mass index (continuous), and smoking status (categories of never/ex) and cigarette pack years smoked (continuous) using linear regression.

***5 quintiles could not formed due to large number of basophils = 0.

−151.1 ml; 95%CI −195.0 to −107.2), and similar associations were seen for granulocytes (highest vs lowest quintile −158.2 ml; 95%CI: −204.0 to −112.4), mononuclear cells (highest vs lowest quintile −116.1 ml; 95%CI: −158.3 to −73.8), and lymphocytes (highest vs lowest quintile −63.3 ml; 95%CI: −101.8 to −24.8). There was no association between either eosinophils or basophils and FVC (Table 3). The sensitivity analyses excluding those individuals with >10 cigarette pack years and cotinine of >3 ng/ml found similar findings such that increased levels total white cell count, granulocytes, mononuclear cells, and lymphocytes were significantly associated with lower levels of lung function, however the effect estimates were up to 30 ml lower in the top quintile.

**Table 3:** Association between categories of blood inflammatory cells and Forced Vital Capacity in non-smoking adults*.

White cell category	Values	Forced Vital Capacity**	
		Coef (ml)	95% CI
**Total white blood cell count (n = 8381)**	1.75–5.255.26–6.26.21–7.157.16–8.358.36–43.65per SD increase	00.90−35.3−93.9−151.1p-trend <0.001−58.2	−44.0 to +45.8−75.1 to +4.6−132.3 to −55.5−195.0 to −107.2−72.0 to −44.4
**Granulocytes (n = 8256)**	0.2–2.952.96–3.73.71—4.44.41–5.45.41–17.75per SD increase	0−17.6−34.0−96.8−158.2p-trend <0.001−56.1	−51.5 to +16.4−75.4 to +7.3−142.6 to −51.0−204.0 to −112.4−70.4 to −41.9
**Mononuclear cells (n = 8256)**	0–0.250.26–0.350.36–0.40.41–0.50.51–5.35per SD increase	0−13.2−15.5−61.5−116.1p-trend <0.001−45.2	−47.4 to +21.1−68.3 to +37.4−104.2 to −18.9−158.3 to −73.8−57.6 to −32.7
**Lymphocytes (n = 8380)**	0.35–1.651.66–1.951.96–2.32.31–2.752.76–33.85per SD increase	0+15.1+17.1−18.2−63.3p-trend = 0.001−20.1	−29.4 to +59.6−22.2 to +56.5−62.1 to +25.7−101.8 to −24.8−30.1 to −10.2
**Eosinophils (n = 2260)**	0–123–45–25per SD increase	0+48.8−20.2−6.8p-trend = 0.77−1.9	−22.6 to +120.2−75.7 to +35.2−84.6 to +71.0−27.8 to +24.0
**Basophils (N = 2260)**	012–6per SD increase	0+33.6+36.7p-trend = 0.11+15.6	−11.2 to +78.5−30.4 to +103.8−4.8 to 36.0

*study population excludes individuals who report a cough, cold or other acute illness in the past few days.

**adjusted for age, sex, height (continuous), race/ethnicity, body mass index (continuous), and smoking status (categories of never/ex) and cigarette pack years smoked (continuous) using linear regression.

***5 quintiles could not formed due to large number of basophils = 0.

There was no association between the FEV_1_:FVC ratio and either total white blood cell count, granulocytes, mononuclear cells, lymphocytes, eosinophils or basophils (data not shown). In addition, there were no significant effect modification by gender of the association between systemic inflammatory cell levels and lung function.

When these associations where examined in a population of smokers, similar associations were observed however the effect estimates were smaller (Table 4). Total white cell count was inversely associated with FEV_1_ (highest to lowest quintile −78.1 ml, 95%CI: 138.7 to −17.6). In addition, granulocytes (highest to lowest quintile −68.3 ml, 95%CI: −132.6 to −4.2), mononuclear cells (highest to lowest −73.0 ml 95%CI: −135.2 to −10.7) were all inversely related to FEV1. In smokers, lymphocytes were not associated with FEV_1_. Similar to non-smoking population, eosinophils and basophils were not associated with lung function.

**Table 4:** Association between categories of blood inflammatory cells and Forced Expiratory Volume in one second in smoking adults*.

White cell category	Values	Forced Expiratory Volume in one second**	
		Coef(ml)	95% CI
**Total white cell count (n = 2790)**	2.4–5.955.96–7.057.06–8.28.21–9.69.61–20.65per SD increase	0−14.7−11.0−63.0−78.1p-trend 0.008−24.6	−69.6 to 40.2−71.0 to 49.0−124.5 to −1.6−138.7 to −17.6−46.2 to −3.1
**Granulocytes (n = 2732)**	0.65–3.353.36–4.254.26–5.15.11–6.256.26–16.8per SD increase	024.2−24.8−28.7−68.3p-trend = 0.018−24.8	−42.3 to 90.6−80.4 to 30.7−112.1 to 54.7−132.5 to −4.2−47.4 to −2.2
**Mononuclear cells (n = 2732)**	0–0.30.31–0.40.41–0.50.51–0.60.61–3.05per SD increase	029.5−20.5−7.6−73.0p-value = 0.021−25.1	−21 2 to 80.3−77.6 to 36.0−72.8 to 57.6−135.2 to −10.7−45.5 to −4.8
**Lymphocytes (n = 2789)**	0.5–1.91.91–2.252.26–2.62.61–3.13.11–14.1per SD increase	048.648.76.4−1.7p-trend = 0.62−4.2	−5.7 to 102.8−2.1 to 99.5−48.2 to 61.0−76.0 to 72.6−23.4 to 15.1
**Eosinophils (n = 798)**	0–123–45–25per SD increase	0−31.4−110.6−83.0p-trend = 0.02−30.1	−123.6 to 60.8−203.5 to −17.7−182.3 to 16.3−64.0 to 3.8
**Basophils (n = 798)**	012–7per SD increase	0−30.4131.6p-trend = 0.5123.2	−118.7 to 57.9−1.0 to 264.2−9.8 to 56.2

*study population excludes individuals who report a cough, cold or other acute illness in the past few days.

**adjusted for age, sex, height (continuous), race/ethnicity, body mass index (continuous), cigarette pack years smoked (continuous) using linear regression.

***no second or third quintiles due to large number of basophils = 0.

## Discussion

This is the first population-based epidemiological study, in men and women in whom recent acute illness has been excluded, to demonstrate an inverse association between the systemic inflammation levels as measured by the total white cell count and lung function using FEV_1_ and FVC. Similar inverse associations were also observed for subgroups of this inflammatory response of granulocytes, and monocytes in both non-smoking and smoking populations, whereas the effect of lymphocytes was only found in the non-smoking study population.

This analysis has a variety of strengths including the high response rate in the NHANES III survey with 86% and 78% of those invited to participate in the questionnaire survey and medical examination doing so [14]. Another strength of this study is the use of systematic measurements of both the white cell count, its constituent cells, blood cotinine to objectively define recent exposure to tobacco smoke, and also physiological measurements of lung function in a well defined population with no knowledge of the hypothesis being tested. In addition, a further strength of this analysis was the use of a large population who provided data on both self-reported smoking history and exposure to recent colds, coughs and acute illnesses.

Cigarette smoking is known to cause acute decreases in lung function [18] and also to promote acute systematic inflammatory responses [19]. We adjusted for this important confounding factor using self reported smoking history and also the objective measure of serum cotinine to identify those individuals who have recently been exposured to cigarette smoke. Similarly, recent coughs, colds and acute illnesses will also impact on the systemic inflammation levels and reduce lung function [12], and the fact that 22% of the eligible population were excluded by this criterion suggests that it has the considerable potential to confound observations of associations of systemic inflammation and lung function. Excluding these individuals from the analysis thus reduces the risk of confounding and strengthens our confidence in the veracity of our data, although it obviously limits the generalisability of this part of the analysis to non-smokers.

We are unable to exclude the possibility of confounding by either exposure to other sources of environmental pollution or sub-clinical infections that may modify either lung function and the cellular inflammatory response. It is also important to consider that data from a cross-sectional study such as NHANES III does not permit temporal relationships to be considered, and therefore we cannot exclude the possibility of reverse causality, i.e. lower lung function leading to an elevated total white blood cell count.

Initial studies of the association between leucocyte count and lung function were reported in 1984 [9]; [10]. Yeung et al [10] used data from two cohort occupational cohorts (n = 3446) and reported an inverse correlation between leucocyte count and both FEV_1_ and FVC. Using data from 1510 men who participated in the Normative Aging Study (a cohort study of healthy men recruited in 1963), Sparrow et al [9] similarly report an inverse association between the peripheral leucocyte count and both FEV_1_ and FVC, after adjustment for age, height and smoking habits. In 1988, Carel et at [11] studied another healthy occupational cohort of 307 men, and again reported similar findings to the first two studies [11].

The only population-based study of both men and women to study the association between the systemic cellular inflammation levels and lung function was by Lewis et al, and did not measure the total white blood cell count, but used leucocyte subgroups to characterise the inflammatory response [20]. In addition, the analysis potentially included those with a recent colds, coughs and acute illnesses as they were not specifically identified and excluded from the analysis. This study of 2369 individuals demonstrated inverse associations between FEV_1_ and systemic neutrophils, monocytes, basophils, eosinophils but not lymphocytes. We reported similar inverse relations for neutrophils (which constitute the majority of granulocytes) and monocytes with both FEV_1_ and FVC. However, we also observed an inverse association between lung function and circulating lymphocytes, but no associations with eosinophils or basophils. One potential explanation for these differences between our data and that of Lewis et al is the method of measuring the differential leucocytes; NHANES III stored frozen blood prior to analysing using a fully automated Coulter hematology analyzer while Lewis at el used a Technicon cell analyzer on all samples within 24 hours. Alternatively, our selection of individuals who had evidence of a recent cough, cold or infection will have created a population that is not directly comparable to that of Lewis et al.

The observation that the systemic neutrophil count is inversely associated with lower lung function is consistent with the hypothesis that airway neutrophil response is important in the pathophysiological processes that drive COPD [21] and asthma [22], although linkage of airway inflammation to disordered airway physiology has been difficult to consistently establish. Similarly, the systemic monocyte count, a precursor of macrophages, was inversely associated with decreased lung function, and again these inflammatory cells are also regarded as having a key role in the development of COPD [23] while eosinophils are inversely associated with lung function in non-smoking men [24]; [25]. However, the inverse association between the systemic lymphocyte response and lung function is a new one and suggests that circulating lymphocyte may also be implicated in the loss of lung function. This is plausible, as histological samples from patients with COPD have demonstrated an increased lymphocyte count compared to those without COPD [26]. It is important to consider that our cross-sectional study design makes it difficult to distinguish between a global increase in the activity of the cellular inflammatory response, and the individual effects of systemic inflammatory cells. These will require more detailed study possibly by observing the parallel airway and systemic cellular inflammatory responses over time.

There has been much interest in the associations between the systemic inflammatory response and lung function with most studies demonstrating an inverse relationship between these variables both in general populations [5]–[8] and in studies that focussed on individuals with COPD [2]–[4]. This has coincided with an increasing appreciation that COPD is a disease with systemic manifestations that contribute to morbidity [27] which require greater understanding and possibly probably targeted interventions to benefit the individual patient. The studies of systemic inflammation may result in enhanced understanding of the pathophysiological mechanisms which can then be used to tailor effective interventions for the varied phenotypes associated with COPD.

In summary, we report for the first time that the total white cell count is inversely associated with both FEV_1_ and FVC in a population of men and women who have not had a recent cold, cough or acute illness. These associations are also observed for circulating granulocytes, lymphocytes and monocytes and lung function. Future studies of inflammation and lung disease should consider utilising these readily available measures of the systemic cellular inflammation levels in addition to the more sophisticated cytokines that are also available. As higher lung function is a predictor of subsequent survival [28]; [29], these associations may contribute to the increased mortality that is associated with a both higher white cell count [29]; [30] and elevated systemic inflammatory status [31] observed in prospective studies.
